# SARS-CoV-2 infection in pediatric population before and during the Delta (B.1.617.2) and Omicron (B.1.1.529) variants era

**DOI:** 10.1186/s12985-022-01873-4

**Published:** 2022-09-08

**Authors:** Haifa Khemiri, Kaouther Ayouni, Henda Triki, Sondes Haddad-Boubaker

**Affiliations:** 1 Laboratory of Clinical Virology, WHO Regional Reference Laboratory for Poliomyelitis and Measles for the EMR, Institut Pasteur de Tunis, University of Tunis El Manar, 13 place Pasteur, BP74 1002 le Belvédère, Tunis, Tunisia; 2 LR 20 IPT 02 Laboratory of Virus, Host and Vectors, Institut Pasteur de Tunis, University of Tunis El Manar, Tunis, Tunisia

**Keywords:** Children, Delta variant, Omicron variant, Symptoms, COVID-19, Prevalence, MIS-C, Immunodepressive, Risk factors

## Abstract

**Background:**

COVID-19, the coronavirus disease that emerged in December 2019, caused drastic damage worldwide. At the beginning of the pandemic, available data suggested that the infection occurs more frequently in adults than in infants. In this review, we aim to provide an overview of SARS-CoV-2 infection in children before and after B.1.617.2 Delta and B.1.1.529 Omicron variants emergence in terms of prevalence, transmission dynamics, clinical manifestations, complications and risk factors.

**Methods:**

Our method is based on the literature search on PubMed, Science Direct and Google Scholar. From January 2020 to July 2022, a total of 229 references, relevant for the purpose of this review, were considered.

**Results:**

The incidence of SARS-CoV-2 infection in infants was underestimated. Up to the first half of May, most of the infected children presented asymptomatic or mild manifestations. The prevalence of COVID-19 varied from country to another: the highest was reported in the United States (22.5%). COVID-19 can progress and become more severe, especially with the presence of underlying health conditions. It can also progress into Kawasaki or Multisystem Inflammatory Syndrome (MIS) manifestations, as a consequence of exacerbating immune response. With the emergence of the B.1.617.2 Delta and B.1.1.529 Omicron variants, it seems that these variants affect a large proportion of the younger population with the appearance of clinical manifestations similar to those presented by adults with important hospitalization rates.

**Conclusion:**

The pediatric population constitutes a vulnerable group that requires particular attention, especially with the emergence of more virulent variants. The increase of symptomatic SARS-CoV-2 infection and hospitalization rate among children highlights the need to extend vaccination to the pediatric population.

**Supplementary Information:**

The online version contains supplementary material available at 10.1186/s12985-022-01873-4.

## Introduction

Severe Acute Respiratory Syndrome Coronavirus 2 (SARS-CoV-2), the virus that causes coronavirus disease 2019 (COVID-19), has spread rapidly around the world since its emergence in Wuhan, China, late 2019 [1, 2]. By January 2020, the virus has been isolated and sequenced [3, 4], revealing close relationships to coronaviruses such as SARS-CoV [5] and MERS-CoV [6]. On March 11, 2020, the World Health Organization (WHO) announced COVID-19 as a pandemic [7] causing 430,257,564 confirmed cases of COVID-19, including 5,922,047 deaths up to 25 February 2022 [8].

SARS-CoV-2 is an enveloped, positive RNA virus [9]. It belongs to the family of *Coronaviridae*, the subfamily of *Orthocoronavirinae,* the genera of *Betacoronavirus* and subgenus of *Sarbecovirus* [10, 11]. It is responsible for severe lower respiratory tract infections in humans [12].

SARS-CoV-2 causes pneumonia, characterized by fever, cough, shortness of breath, and bilateral infiltration on chest imaging [2, 12]. It can also induce fatal lung damage, multiple organ failure and death [9, 13]. The clinical manifestations can be classified into four severity of illness categories: asymptomatic, mild, moderate and severe clinical [14]. In contrast with adults who can develop the four types of clinical manifestations, children are mainly asymptomatic or present mild infection and, in some cases, they can develop severe post-COVID-19 manifestations such as Kawasaki-like symptoms [15–18]. After a year into the COVID-19 pandemic, new variants emerged and spread rapidly across the continents [19]. In the United Kingdom, the B.1.617.2 Delta variant, initially identified in India in October 2020, spreads rapidly through schools [20]. Children seem to be the most affected category [19, 21, 22]. The B.1.1.529 Omicron variant first detected in South Africa, in 2021, appeared to be more contagious than the Delta variant, associated with a significant increase in the number of pediatric and adults SARS-CoV-2 infections [23–28].

This review gives an overview of SARS-CoV-2 infection in children before and after B.1.617.2 Delta and B.1.1.529 Omicron variants emergence. We analyzed current knowledge on the prevalence, clinical manifestations and complications among immunocompetent and immunodepressive children as well as risk factors for the severity of COVID-19.

## Methods

**Fig. 1:**
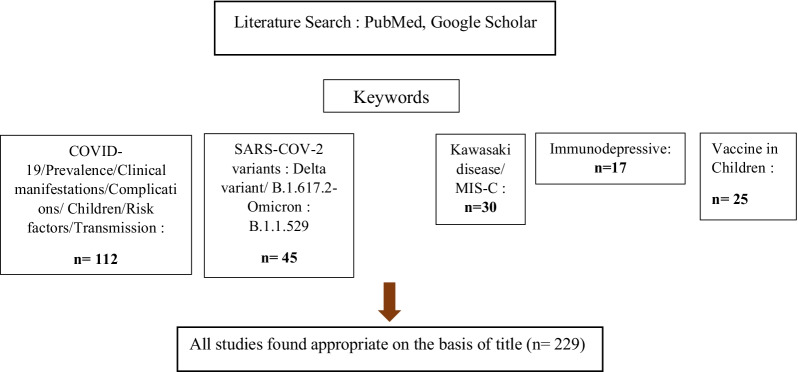
The literature search on COVID-19 in children before and during the Delta and Omicron variants era

Original research studies published on COVID-19/SARS-CoV-2 among children in English, between January 2020 and July 2022, were identified using PubMed, Science Direct and Google Scholar. The search used combinations of the keywords “COVID-19,” “SARS-CoV-2,” “clinical manifestations” “prevalence”, “Transmission”, “pediatric population,” “child”, “SARS-CoV-2 variants”, “Delta variant”, ”B.1.617.2”, “Omicron variant”, “B.1.1.529”, “Risk factors” and “COVID-19 vaccines” (Fig. 1). In addition, the reference lists of the retrieved articles were checked for other relevant articles. Moreover, 229 references were considered relevant to the aim of this review. An additional table file shows more details about these references (see Additional file 1: Table S1).

## Epidemiology of COVID-19 in infants and children

### Epidemiology of COVID-19 in infants before the Delta and Omicron era

At the beginning of the COVID-19 epidemic, the available data during the period February 26, 2020, to June 10, 2020, suggested that the infection occurs more frequently in adults and seems to be unusual in infants (1.7–2% of the diagnosed cases of COVID-19) [29, 30]. On February 11, 2020, the Chinese Center for Disease Control and Prevention (China CDC) showed that among 72,314 cases less than 1% and 1% of the cases were in children younger than 10 years and 10 to 19 years, respectively [31]. In the United States, data concerning 149,760 laboratory-confirmed COVID-19 cases obtained between February 12 and April 2, 2020, showed that 1.7% were in children aged less than 18 years [32]. From March 5 to April 8, 2020, the Chicago Department of Public Health reported that among 6369 laboratory-confirmed cases, 1.0% were children aged 0–17 years [30].

Afterward, it appeared that the true incidence of infection in infants was underestimated [33]. A retrospective study based on Nationwide case series of 2135 COVID-19 pediatric patients (< 18 years) with COVID-19 reported to the China CDC, from January 16, 2020, to February 8, 2020, indicated that children of all ages appeared to be susceptible to COVID-19 [33]. In this study, 731 (34.1%) children had laboratory-confirmed COVID-19. The Center for Disease Control and Prevention (CDC) showed an increase in the rate of COVID-19 pediatric cases, from March to July 2020, in the USA: 7.3% of all COVID-19 cases compared to 1.7% of COVID-19 pediatric cases obtained during the period 12 February to 2 April [34, 35]. It seems that children and adults acquired infection at similar rates but develop different clinical manifestations [33]. In China, several studies including population varied from 731 to 1391 children showed only a few numbers of infants (1–2%) developed symptomatic forms [31–36]. Also, a systematic review conducted by Bhuiyan et al. [37] showed that among 1214 confirmed COVID-19 pediatric cases younger than five years nearly half of the cases were asymptomatic.

**Fig. 2:**
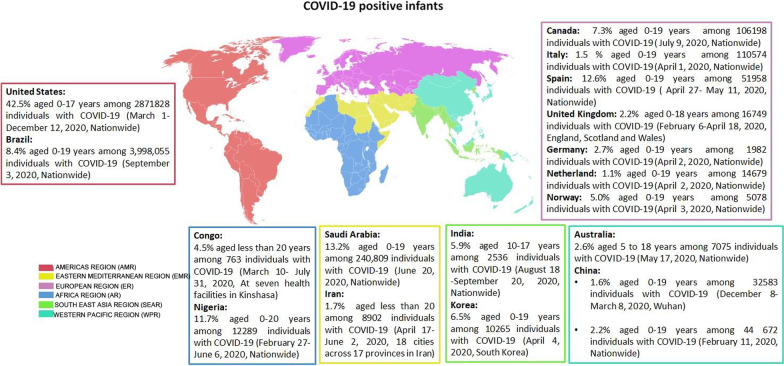
Prevalence of positive COVID-19 infants in different regions of the World Health Organization (WHO): AMR colored in red, EMR colored in yellow, ER colored in purple, AR colored in blue, SEAR colored in green and WPR colored in blue light

Globally, the prevalence of COVID-19-positive infants was underreported. The number of COVID-19-positive infants was reported only in a few studies in which, it is indicated among other positive cases [38, 39]. Figure 2 shows the prevalence of COVID-19-positive infants in different regions of the world based on the reported data before the emergence of the delta and omicron variants. The prevalence of COVID-19 in infants varied from country to country (1.1–22.5%) (Fig. 2). The highest percentages of COVID-19 positive infants were from United States (22.5%), Saudi Arabia (13.2%), Spain (12.6%) and Nigeria (11.7%) [40–43]. Lower percentages were found in Brazil (8.4%), Canada (7.3%), Korea (6.5%), India (5.9%), Norway (5.0%), Congo (4.5%), Germany (2.7%), Australia (2.6%), United Kingdom (2.2%), China (1.6–2.2%), Iran (1.7%), Italy (1.5%) and Netherland (1.1%) [37, 39, 44–48]. These differences may be explained by the possible country-level influence on COVID-19 such as the testing strategy of suspected cases, the original population structure of each country, and whether the control measures (social distance, wearing masks, washing hands, etc.) were strictly followed by the public or not and also by the period of the state of the epidemic. However, it is necessary to point out that the true prevalence of COVID-19 in infants remains underestimated due to the lack of stated data and the high frequency of asymptomatic forms [49].

### Epidemiology of COVID-19 in infants during the Delta and Omicron era

#### During Delta era

Since its emergence in December 2020, the delta variant (B.1.617.2) seems to affect a large proportion of the younger population with the appearance of clinical signs similar to those presented by adults [21, 22, 50]. Main data was obtained from the UK, after April 2021, where this variant became a dominant strain and was spread rapidly through schools [20]. Public Health England confirmed an increased number of outbreaks and clusters at primary and secondary schools from May to June 2, 2021 [20]. Reports from different areas such as Bolton indicated that cases were growing fastest among school-age children, with an increased number of cases at any point during the pandemic [51]. Also, data from the Office for National Statistics of UK for the weeks of 29th May and 1st June, showed an increase in the numbers of cases in school children aged 7–11 years [20, 52]. In the United States, it was suggested that the delta variant was highly transmissible in indoor sports settings and households, which might lead to higher attack rates among exposed persons especially children aged 5–19 years [53]. Overall, in the epidemiological analysis of COVID-19 in infants during the Delta variant era, there was a significant increase in pediatric cases and hospitalizations [54–56]. Data suggested that these increases are related to increased Delta COVID-19 incidence rather than increased Delta virulence in infants [54, 55, 57].

#### During Omicron era

**Fig. 3:**
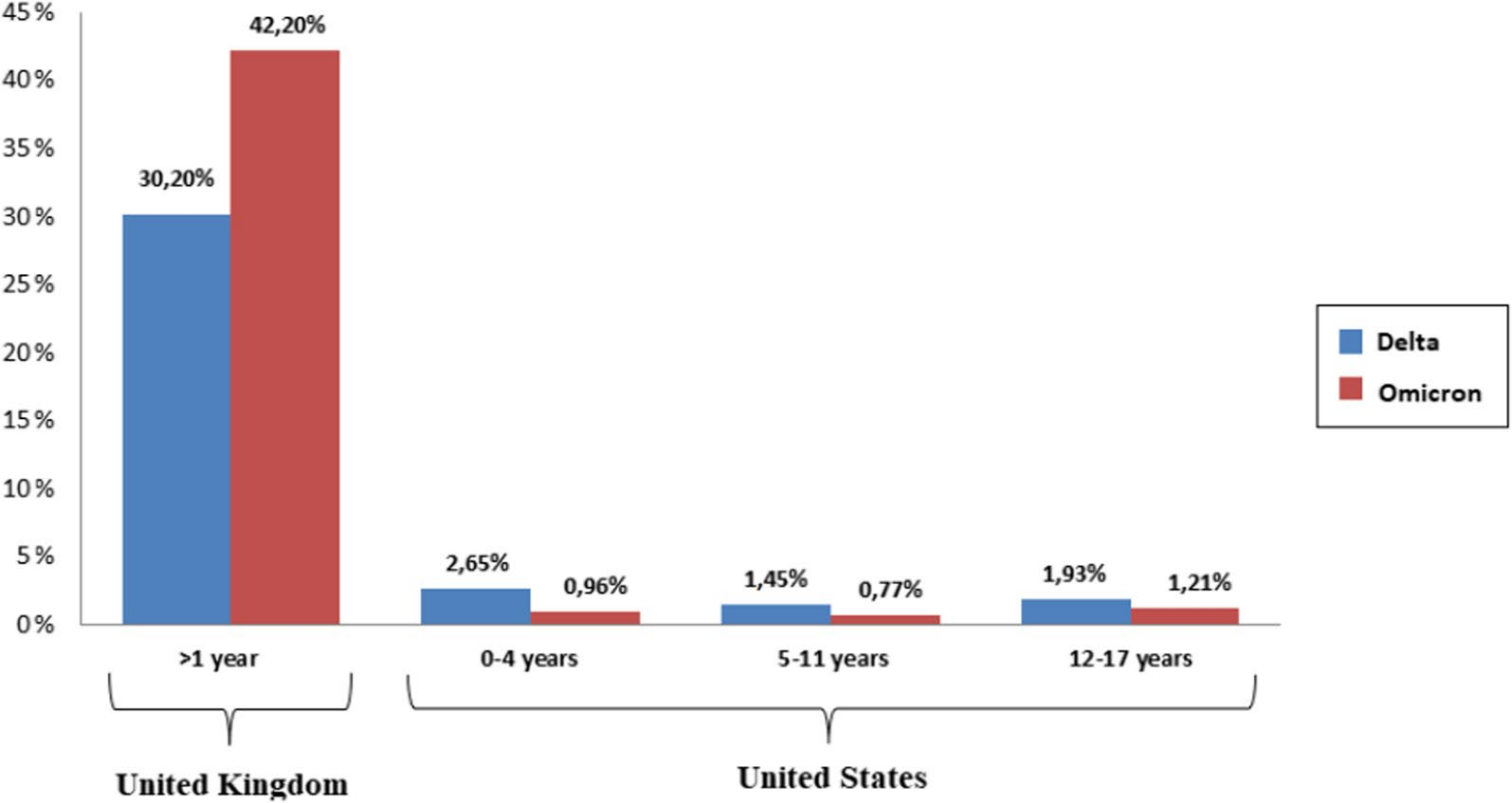
Prevalence of hospital admission of COVID-19 infants before and after the emergence of omicron variant

The emergence of the Omicron variant was also linked to an increasing number of SARS-CoV-2 infections and hospital admissions of infants and adults. Available data showed rising in pediatric SARS-CoV-2 hospitalizations in many countries [23–28]. In the UK, according to the data from the COVID-19 Clinical Information Network study, the proportion of infants aged less than 1 year admitted to hospital was 42.2% (period from 14 December 2021 to 12 January 2022), much higher than earlier in the pandemic (Fig. 3) [23, 24]. However, reports from Scotland, the US, South Africa, and England showed lower rates of hospitalization following Omicron infection compared with the Delta variant infection [58–61]. The national data from a Scotland cohort analysis suggested that Omicron is associated with a two-thirds reduction in the risk of COVID-19 hospitalization when compared to Delta [59]. In the US, a retrospective cohort study conducted on 577,938 patients during the period when Delta and Omicron variants predominate showed that the outcomes in children under 5 years old are mildest after the emergence of the Omicron variant [61]. The risk for hospitalization and the risk for emergency department admission that occurred after the emergence of the Omicron variant was one-third (Fig. 3) and less than one-fifth of that during the Delta variant period, respectively [61]. These findings were also observed for children aged 5–11 and 12–17 years old (Fig. 3) [61]. The increased rate of infections and hospitalizations caused by the Omicron variant compared with the Delta variant seems to be attributed to differences in the prevalence of the virus variants.

## Transmission dynamic among infants and adults

### Transmission dynamic Before Delta and Omicron era

The need to understand the impact of children in the SARS-CoV-2 pandemic sighted researchers to study the transmission dynamics of SARS-CoV-2 between infant-infant and infant-adult. From February 2020 to January 2021, the obtained data were controversial. Many studies reported low transmissibility of SARS-CoV2 among children and also from children to adults [36, 38, 62, 63]. For instance, when investigating SARS-CoV-2 transmissibility in 23 clusters, Maltezou et al. showed that the transmission of infection within families including children occurs more frequently from adult to child [63]. Low transmissibility in children was also reported by other studies conducted in China and United States, which it has shown that young children often acquired infections through community sources [35, 37]. Bhuiyan et al. [37], found that among 1214 children younger than five years, the majority (> 95%) had a community source of infection. However, in the same period, other studies suggested an important role of children in the transmission dynamics of SARS-CoV-2 [30, 64]. A household transmission pattern study, in the United States, demonstrated that children were the potential source of further transmission in approximately 20% of households, with possible child-to-adult transmission in 20% and possible child-to-child transmission in 17% [64]. Also, Mannheim et al. suggested both child-to-child and child-to-adult transmission is estimated at 13% [30]. In India, based on the contact-tracing data from the Indian states of Tamil Nadu and Andhra Pradesh by 1 August 2020, Laxminarayan et al. [1] showed that the transmission occurs more frequently among children who contact cases from their own age. The enhanced infection risk among individuals exposed to similar-age cases was also apparent among adults [1]. The authors concluded that children may play a key role in the transmission dynamics of SARS-CoV-2 in middle and low incomes countries where social, cultural, and economic conditions favor close contact between children and also with adults [1, 65].

### Transmission dynamic during the Delta and Omicron era

#### During Delta era

The emergence of novel variants much more transmissible raised questions about the change in the relative risk of SARS-CoV-2 child–adult transmissibility [66]. Loenenbach et al. [67] 2021 showed that the susceptibility and infectiousness of children having the SARS-CoV-2 B.1.1.7 variant are substantially higher compared with the pre-variants of concern (VOC) period (39% vs. 7.9%). The SARS-CoV-2 B.1.617.2 variant seems to be around 60% more transmissible than the SARS-CoV-2 B.1.1.7 variant [68, 69]. It is spreading rapidly, particularly through primary and secondary schools as well as indoor sports settings and households, which might lead to higher attack rates among children [20, 21, 53].

#### During Omicron era

A rapid increase in COVID-19 cases was reported from the beginning of December 2021, as a result of the Omicron variant spreading. The transmission dynamics of this new variant was assessed by an in-silico analysis. Data from this study showed that the infectivity of Omicron might be more than tenfold higher than that of the original virus and approximately twice as high as that of Delta [70]. According to reported data, it is estimated that the Omicron variant infects six times as many people as Delta over the same time and that the Rt of Omicron is 1.4- to 3.1-fold higher than that of Delta [25, 26, 71]. In Gauteng South Africa, Viana et al. [28] estimated that Omicron had a growth advantage of 0.24 per day over Delta, corresponding to a 5.4-fold weekly increase in cases compared to Delta. Data on the rate in children in the US showed that the rate of infected children admitted to hospital from 26 December 2021 to 1 January 2022 was nearly double the rate reported in early December before the Omicron variant began to take hold [27]. Current data shows that the Omicron variant is more contagious than the Delta variant. However, evidences from other international studies are needed to highlight the role of SARS-CoV-2 variants in increasing outbreak size.

## Incubation period

The incubation period is the duration from the exposure of the pathogen to the onset of symptoms. Studies report a median incubation period of 5 to 7.5 days, before the emergence of the B.1.617.2 variant. Shen et al. [72] showed that the median incubation period was 7.5 days in six children ranging from 1 to 16 days. In another study, the median incubation period of children was 5 days (ranging from 3 to 12 days) [73]. It was shown that the incubation period of SARS-CoV-2 differs from variant to another (Table 1). The incubation period for the Delta variant, to the best of our knowledge, was only reported in four studies from China and Japan [74–77]. It was significantly shorter (3.7, 4.0 and 4.4 days) than for non-Delta strains (4.9–6.0 days) [74–76]. However, in another study, a longer incubation period was reported (6.0 days) [77]. After the emergence of the Omicron variant, preliminary information suggests that the median incubation period may be shorter at around 2–3 days in children and adults [78].

**Table 1:** Incubation period, rates of emergency department visit and intensive care unit admission compared during the period before and after Delta and omicron emergence

	Before Delta and omicron emergence	After Delta emergence	After Omicron emergence	References
Median Incubation period	5–7.5 days	3.7–6 days	2–3 days	[77, 78]
*Emergency department visit*				
0–4 years	NI	21.01%	3.89%	[61]
5–11 years	NI	12.62%	3.60%	[61]
*Intensive care unit admission rate*				
Under 1 year	NI	14.00%	9.90%	[23]
< 18 years	26.5% 0.10%	23.2% 1.40%	NI NI	[120] [54]

## Common clinical manifestations

### Before the Delta and Omicron era

At the onset of the SARS-CoV-2 pandemic up to May 2021, compared to adults, children get less COVID-19 and had less severe cases [33, 48, 62–64]. Unlike adults, infants develop more likely asymptomatic [33, 36, 72, 79–83] or mild forms [16, 31, 33, 36, 64, 84, 85]. Symptomatic children reported fever and dry cough as the most common symptoms. They were reported at 36–73% and 19–54% of pediatric cases, respectively [32, 35, 48, 66, 67, 79, 86–93]. The body temperature varied mainly between 37.5 and 38.5 [86]. These symptoms can be accompanied by headache, fatigue, myalgia, nasal congestion, sneezing, [28, 48, 64, 68, 69, 79, 86, 88, 89, 94]. Headache was reported in almost 25–79% of cases [48, 64, 70, 71].

Regarding nasal congestion, some studies, in the USA and China reported an important proportion of 50–68% of pediatric cases with rhinorrhea [64, 94]. In contrast, other studies based on a limited number of cases, reported fewer proportions ranging from 2.8 to 16% of cases with rhinorrhea and nose congestion [86, 87, 89, 91, 95].

While adult patients rarely developed intestinal signs, symptoms such as nausea, vomiting, abdominal pain, constipation, and diarrhea are frequent among pediatric populations [96, 97]. A wide variety of manifestations can appear before, with, or after the development of respiratory symptoms [67–69, 79, 96, 98–100]**.** Anorexia or poor appetite was frequent (34–67%), followed by diarrhea (2–32.5%), vomiting (1–28.3%), abdominal pain and discomfort (1–11.9%) and nausea (1–11.1%). [9, 79, 96, 99, 101–110].

Compared with symptomatic adults, children were less likely to report loss of taste and loss of smell [48, 64] and more likely to report sore throat [64, 83, 86, 88, 89]. In Taiwan, up to April, 7, among 24 children, 12.5% had a loss of smell and/or taste [48]. Other studies reported also the development of sore throat. According to Laws RL and colleagues in a series of 19 children, 68% had presented sore throat [64]. However sore throat seems low in other studies: 1 case in a total of 20 [88], 2 cases of sore throat among 31 [89].

### Clinical manifestations during the Delta and Omicron era

#### During Delta era

The Delta variant infection appeared to cause a similar common outcome [56, 111]. A retrospective study in China investigated the clinical features of delta-infected patients and non-delta ones and showed that cough and fever are still predominant signs, but gastrointestinal symptoms have become much less frequent [111]. In South Korea, a retrospective study showed that there was no significant difference in COVID-19 symptoms in delta and non-delta patients except for the lower frequencies of rhinorrhea (25% vs. 10.5%, P = 0.003), nasal stuffiness (34.8% vs. 15.4%, P = 0.001) and sore throat (23.9% vs. 12.6%, P = 0.02) [56]. Additionally, it was shown that there was no statistically significant difference between the two groups in the frequency of pneumonia (2.2% vs. 0.7%, P = 0.56) and hospital transfer (5.4% vs. 2.1%, P = 0.27) [56].

#### During Omicron era

The same common manifestations were observed with Delta and Omicron variants. A study comparing the outcome of COVID-19 infection in pediatrics and adults before and after the emergence of the Omicron variant showed that both Delta and Omicron variants induce relatively stable outcomes [61]. However, higher manifestations rates were associated with the Omicron variant due to its higher infectivity compared to the other variants [61].

## Complications of COVID-19

### Complications before the Delta and Omicron era

Before the emergence of the Delta variant, complications among positive COVID-19 children cases were less frequent. They were especially reported among patients with underlying medical conditions. Required hospitalization was in 11% to 60% of cases and admission to the Intensive Care Unit (ICU) in almost 10% of cases, the majority presented medical underlying conditions [32, 93, 112, 113].

Severe cases presented hypoxia, tachypnea and oxygen saturation less than 92% [33, 93]. Critical cases can rapidly progress into acute respiratory distress syndrome or respiratory failure and present shock, encephalopathy, myocardial injury, heart failure, coagulation dysfunction, acute kidney injury and Organ dysfunction [33, 87, 94].

### Complications during the Delta and Omicron era

#### During Delta era

After the emergence of the Delta and Omicron variants of SARS-CoV-2, available information reported high hospitalization rates among children [19, 114–116]. Furthermore, a study conducted in Scotland and England showed that the risk of COVID-19 hospitalization was approximately double in those with Delta VOC compared with Alpha VOC and that, particularly in those with comorbidities [117, 118]. However, other studies showed that the increased rate of hospitalization during the Delta emergence was not associated with a severe outcome with a similar rate of Intensive Care Unit admission (Table 1) [69, 119, 120]. In Japan, the intensive care unit admission rate was higher during the Delta VOC era than in the pre-Delta VOC era (1.4% [n = 5] vs. 0.1% [n = 1], P = 0.006), among pediatric patients with COVID-19 (Table 1), but no patient in either group died or required mechanical ventilation or extracorporeal membrane oxygenation [54]. According to these data, it appears that the Delta variant does not cause worse clinical outcomes compared to prior lineages. Furthermore, a retrospective study investigating imaging characteristics of children infected with the Delta variant, showed that the imaging-based severity was significantly milder than that in cases in 2020 [121].

#### During Omicron era

For the Omicron variant, based on a multicenter nationwide database in the US, no major changes in hospitalization were observed during the two-week that immediately preceded the emergence of the Omicron variant (11/16/2021–11/30/2021) and the 10-week Delta variant period before it (9/1/2021–11/15/2021) [61]. A lower rate of emergency department visits was found among children and adults compared to the Delta variant period (Table 1) [61]. In the UK, it was shown that the rate of admission to intensive care was less than that detected during the delta period (Table 1) [23].

## Immunodepressive children

### Immunodepressive children before the Delta and Omicron era

As COVID-19 can trigger complications of an underlying disease, patients with immune deficits need to be monitored carefully during the COVID-19 pandemic [122]. Nevertheless, at the beginning of the pandemic, studies about COVID-19 among Primary Immunodeficiency (PID) patients were controversial. According to the Joint Forces of International Societies for Immunodeficiencies, most patients present a mild disease [122, 123]. For instance, In Iran, a case report of an eight-year-old boy who had Specific Antibody Deficiency (SAD), presented wet cough and rhinorrhea [124]. In general, patients with severe disease evolution, additionally, have comorbidities or complications of their immunodeficiency [122, 123].

Other studies considered that patients with immunodeficiency are at increased risk of developing severe COVID-19 [125–127]. PID patients presented: shortness of breath, a drop in oxygen saturation, respiratory distress, tachypnea, seizure, cardiomegaly, cardiac, and pulmonary arrest, requiring hospitalization and admission to intensive care units [128–130]. For instance, among an international series of 94 COVID-19 PID patients, 18 were admitted to intensive care units, and nine patients died [129]. Another study reported up to 50% hospitalization rate among PID requiring mechanical ventilation [130–132]. Fatal outcomes were also reported among PID and Secondary Immunodeficiency (SID) patients. It was estimated up to 35.3% and 44% respectively [130].

It was expected that prolonged carriage of SARS-CoV-2 is possible such as in the case of viruses belonging to the family of Picornaviridae: poliovirus [133], non-polio enteroviruses [134, 135] and cosavirus [136]. Data reported shedding of infectious SARS-CoV-2 and genomic RNA by PID occurred for many months [137]. They excreted the virus up to 120 days after initial detection (70–120 days) [138]. In these cases, Primary Immunodeficient (PIDs) and Secondary Immunodeficient (SIDs) patients, may constitute an increased risk of transmission to the community with the persistence of a reservoir for the virus and the potential emergence of mutated strains.

### Immunodepressive children during the Delta and Omicron era

The available data on immunocompromised patients infected with the delta variant is limited. According to the study of Hadjadj and colleagues, the emergence of SARS-CoV-2 strains like the Delta variant (B.1.617.2) with increased viral form and immune escape potential, raises concerns among immunocompromised patients [139, 140]. Recently, a new variant of concern (VOC) called Omicron was first identified on November 24, 2021, in an immunocompromised patient from Johannesburg, South Africa as a consequence of prolonged replication among PID patients [141–145]. However, no more data has been published on the behavior of omicron in severely immunocompromised individuals [146].

## Kawasaki-like and Multisystem Inflammatory Syndrome COVID-19 manifestations

SARS-CoV-2 can induce a systemic inflammatory response affecting multiple organs, with severely affected lungs. The extrapulmonary manifestations can include systemic vasculature, similar to Kawasaki disease (KD) or Multisystem Inflammatory Syndrome [18, 147]. KD is an acute systemic inflammatory disease of medium- and small-sized vessels that mostly involve children under 5 years old [148–152]. In the acute phase of the disease, patients might have haemo-dynamic instability, a condition known as Kawasaki Disease Shock Syndrome (KDSS) [153]. The cause of KD is unknown, but it is generally accepted that viral agents can trigger the disease [18]. The diagnosis is clinical and based on a combination of fever for at least five days and generalized inflammation involving lymph nodes, skin, and mucous membranes [147, 154, 155]. It can also induce conjunctivitis, coronary artery dilation/aneurysms and bilateral bulbar, [18, 156, 157]. Several cases have shown that SARS-CoV-2 stimulates an immune reaction mimicking KD [87, 156, 158–166, 185]. Association to COVID-19 was mainly confirmed by serology test since clinical manifestations occur during the post-COVID-19 period in Children [87, 147, 156, 159–166]. Children can present classical form of the Kawasaki disease or incomplete (atypical) form [156, 158–160, 162, 167–175].

Multisystem Inflammatory Syndrome in Children (MIS-C) is a pediatric hyperinflammation disease (cytokine storm) that was associated with SARS-CoV-2 infection [18, 158]. In general, it manifests 2–4 weeks following their initial COVID-19 infection as a consequence of an excessive immune response [167]. The clinical presentation of MIS-C includes high fever, inflammation and multi-organ dysfunction including gastrointestinal, cardiovascular (high frequency of myocarditis up to 80%), hematological, mucocutaneous, and respiratory [18, 168–173]. MIS-C appears to share clinical features with KD, toxic shock syndrome and macrophage activation syndrome (MAS) [18, 170]. Despite this, MIS-C differs from KD in the involvement of greater age, gastrointestinal, myocarditis and/or cardiogenic shock, heart failure requiring inotropic support and circulatory assistance [18, 170]. On the other hand, comorbidity was associated with MIS-C manifestation including asthma and overweight. [175].

## Outcomes

In the pediatric population, disease duration is relative to the severity of the disease. Indeed, the duration is over 10 days across all patients and over 20 days in critically ill patients [13, 88, 89, 91, 94].

The length of hospital stay has varied between different studies: the minimum and maximum averages reported have been 8.3 and 15.3 days respectively [72, 91]. In the case of hospitalization, the mean time was 14 days [72, 86]. Qiu et al. [86] concluded that patients with the moderate clinical type spent more days in hospital compared with those with a mild clinical type. Delta variant patients seem to have a significantly longer median (5.7) hospital length of stay [176]. In general, children who are admitted to PICU recovered without any adverse outcomes [87]. Fatal outcomes were also reported, generally in patients with underlying health conditions [32, 92, 177].

## Factors impacting lower susceptibility of children to COVID-19 infection

There are several hypotheses on the mechanisms underlying the lower susceptibility of children to COVID-19 infection than adults [178, 179]. First, there is a lower expression of the Angiotensin-Converting Enzyme 2 (ACE2) receptor to which the virus would bind to enter cells [180]. ACE2 is a receptor for SARS-CoV-2 and the key region responsible for the interaction [106, 181]. Differences in the distribution of ACE2 in the developing phase of childhood are a possible reason for milder SARS-CoV-2 infection [29]. Second, an “immaturity” and consequently loss of functioning of the ACE2 receptors, makes it difficult, for the virus to enter the body [182]. Third, kids present a great expression of the innate immune response, which is more effective against this virus than adults [179, 183]. Children seem also to present a more efficient immune response due to the stimulation given by typical age and vaccinations [184]. According to the literature, some vaccines such as HBV, Tetanus, Measles, Mumps, Rubella and BCG may have a protective effect against COVID-19 [185–187].

## Factors impacting the severity of COVID-19 in children

### Before the Delta and Omicron era

There are many risk factors associated with the progression and severity of COVID19 in children such as age, male gender, ethnicity, comorbidity, coinfection and virus type [29].

In the United States, Bandi S and colleagues reported that the mean age of COVID-19–positive children was significantly higher than those testing negative (9.72 vs. 4.85 years) [188]. In another study, severe and critical cases were reported mainly in children aged less than 1 year [36]. However, DeBiasi RL and colleagues showed that adolescents and young adults were more commonly critically ill than younger children [189].

Ethnicity was examined in the study of Bandi et al. [188]. African American children had a significantly higher rate of positive tests for COVID-19: 6.8% versus 1.7% of white children. Among 58 children, in the United Kingdom, race (black or Asian) was described as a risk factor for COVID-19 [190].

Male gender is a risk factor for severe coronavirus disease in adults [191]. A predominance of boys was reported in all age subgroups among 2490 pediatric cases of COVID-19 in a series in the United States [32]. In a cross-sectional study of 48 children with COVID-19 admitted in the USA and Canadian intensive care units (ICUs), 52% were boys [192].

Underlying medical comorbidity may be a risk factor for severe disease in childhood [32, 80, 88, 92, 177, 192, 193].

The rate of patients with preexisting morbidity, developing severe COVID-19 manifestations, varied between 23 and 83% [193]. Among underlying diseases, chronic lung disease was the most common followed by cardiovascular disorder and immunosuppressive disease [32]. Malnutrition, cancers and kidney diseases were also implicated [178]. Respiratory syncytial virus and *Enterobacter aerogenes* were implicated [87, 194].

### During the Delta and Omicron era

#### During Delta era

The emergence of the delta variant B.1.617.2 confirmed the impact of virus strains on the severity of the disease. B.1.617.2 was considered as the most dominant SARS-CoV-2 variant before the appearance of the Omicron variant and it was rapidly spread around the world [195, 196]. The Delta variant harbors different mutations in the Spike gene that helped the virus to be more transmissible, escape immune response, easily break into cells and efficiently replicate (T19R, L452R, T478K, D614G, P681R, and d960N, G142D, R158G with deletions at positions 157 and 158) [195, 197, 198].

For instance, D614G interrupts certain molecular interactions in the spike protein facilitating virus propagation. L452R can enhance the interaction between ACE2 and the spike protein, which makes the virus more susceptible to infect cells. T478K is unique to the delta variant. Its role has not yet been described and like L452R, it may help enhance the maintenance of spike proteins on ACE2 [197, 199, 200].

#### During Omicron era

After this wave, a new variant B.1.1.529 was first reported to the WHO in South Africa on November 24, 2021, called Omicron [141]. This variant contains a large number of mutations. It included more than 30 mutations in the spike protein alone compared to delta variant mutations in addition to others in proteins such as NSP12 and NSP14 which enhanced the viral replication [145, 195, 201–203]. If vaccine coverage is still insufficient in any region of the world, it will be possible in the future that other varieties will emerge with significant changes in transmissibility, infectivity and pathogenicity.

## SARS-COV-2 vaccines in children

After the emergence of the Delta variants, children of all ages seem to be more vulnerable to develop severe cases. Thus, they should benefit from vaccination to protect themselves from severe and long-term consequences and, also, to restrain virus circulation among the population. Two types of mRNA vaccines, Pfizer-BioNTech (BNT162b2) and Moderna, were allowed for children [204–209]. The Pfizer vaccine was authorized, in May 2021, for children aged more than 12 years, and in December 2021, for children aged 5–11 years [205]. In July 2021, the European Medicines Agency (EMA) authorized the Moderna vaccine for those aged 12–17 years, while vaccination of kids aged 5–11 years is still under-investigation [210]. Both vaccines have been applied, in children over the age of 12, in American, European, African and Asian countries [211–222]. While only Pfizer has been implemented for those aged 5–11 years, in Italy, the United States, Israel, United Kingdom, Germany, Ireland, Poland, France, Spain and Denmark. [210–213, 218, 223–225]. Several studies have shown that vaccines were safe and effective in reducing virus transmission and protecting them against re-infection with SARS-CoV-2 [205, 226–228]. However, they may cause mild to moderate adverse reactions, such as fever, fatigue and irritability, with a low risk of myocarditis, especially for children aged 5–11 years [205, 225–227].

## Conclusion

The pediatric population constitutes a vulnerable group that requires particular attention during the SARS-CoV2 pandemics and especially with the emergence of more transmissible and virulent SARS-CoV-2 variants. Although children are less susceptible to COVID-19, and the clinical picture in childhood is often distinct from that in adults, in both age groups chronic underlying medical problems, immunodeficiency and virus virulence can predispose to severe disease. In contrast with adults, in whom older age is an independent risk factor for severity and mortality, very young age is considered a risk factor for severity in children, The care of children with allergies or immune conditions is being adapted to the current situation, with more remote working and guiding children to reduce the likelihood of infection in children who would be deemed at higher risk of severe COVID-19 disease [122]. The current COVID-19 pandemic might also pose a risk to pediatric patients with secondary immunodeficiencies, such as patients on immunosuppressive therapy for autoimmune or severe allergic diseases [122]. With the emergence of the Delta and Omicron variants, infection in children seems to be more frequent and severe. The increase of symptomatic and severe SARS-CoV-2 infections highlights the need to extend vaccination to the pediatric population. The Pfizer and Moderna vaccines were authorized for children since they were found to be safe, immunogenic, and efficacious.

## Supplementary Information

**Additional file 1: Table S1** General information about the included studies and their references.

## Data Availability

The present study was a systemic review based on published original research articles. All data are publicly available within the manuscript. We have accessed the published articles.

## Abbreviations

ACE2: Angiotensin-converting enzyme 2
COVID-19: Coronavirus disease 2019
WHO: World Health Organization
ICU: Intensive Care Unit
KD: Kawasaki Disease
KDSS: Kawasaki Disease Shock Syndrome RNA: Ribonucleic Acid
MAS: Macrophage Activation Syndrome
MIS-C: Multisystem Inflammatory Syndrome in Children
NSP12: Non Structural Protein 12
NSP14: Non Structural Protein 14
HBV: Hepatitis B Virus
PICU: Pediatric Intensive Care Unit
PID: Primary Immunodeficiency
SAD: Specific Antibody Deficiency
SID: Secondary Immunodeficiency
VOC: Variants of Concern
USA: United States of America
