# Supplementary material for: SARS-CoV-2 infection in pediatric population before and during the Delta (B.1.617.2) and Omicron (B.1.1.529) variants era
Source: Virol J. 2022 Sep 8;19:144. doi: 10.1186/s12985-022-01873-4 (PMC9452867; doi:10.1186/s12985-022-01873-4)
Supplement: Supplementary file 1 — Additional file 1: Table S1 General information about the included studies and their references. [file 12985_2022_1873_MOESM1_ESM.pdf]

Table 1: General information about the included studies and their references

Authors/ Reference	Date of investigations	Town/Country	Population	Main Idea
**Laxminarayan R et al [1]**	March – July 2020	Indian states	575,071 individuals	Epidemiology and transmission dynamics of COVID-19
**Rathore JS and Ghosh C [2]**	March-May, 2020	India	ND	SARS-COV-2 origin and classification
**Zhou P et al [3]**	December 2019-January 2020	Wuhan, China	2,761 peoples	characteristics of SARS-COV-2
**Zhu N et al [4]**	December 2019- January 2020	China	4 respiratory samples	Detection and Isolation of a Novel Coronavirus
**Drosten C et al [5]**	March 3 to 9, 2003	Germany	3: 1 patient and 2 contacts	Isolation and characterization of a novel coronavirus
**Zaki AM et al [6]**	June 13, 2012	Saudi Arabia	1 sample	Isolation of a novel coronavirus from a man with pneumonia
**World Health Organization [7]**	March 11, 2020	In the World	Global population	World Health Organization (WHO) announced COVID-19 as a pandemic
**World Health Organization [8]**	February 25, 2022	In the World	430 257 564 confirmed cases of COVID-19	Number of confirmed cases and deaths
**Huang C et al [9]**	Jan 2, 2020	Wuhan, China	41 patients	Clinical features of patients infected with 2019 novel coronavirus
**Wang H [10]**	January 12, 2020	China	ND	The genetic sequence, origin, and diagnosis of SARS-CoV-2
**Wassenaar T m. and Zou Y [11]**	January-February 2020	China	ND	classification of betacoronaviruses and identification of Traditional Chinese Medicine as potential origin of zoonotic coronaviruses.
**Lotfi M and Rezaei N [12]**	December 2019-March 2020	Iran	ND	SARS-CoV-2: A comprehensive review from pathogenicity of the virus to clinical consequences
**Zhang T et al [13]**	February 3to 17, 2020	Tianjin, China	3 infected children	Detectable SARS-CoV-2 viral RNA in feces of three children during recovery period of COVID-19 pneumonia
**World Health Organization [14]**	May 27, 2020	In the World	Children under 18 years old	Clinical management of COVID-19: interim guidance
**Jahangir. M et al [15]**	December 1, 2019- April 9, 2020	In the World	Pediatric population	Clinical manifestations and outcomes of COVID-19 in the pediatric population

**Rezaei N [16]**	April 4, 2020	Iran	Children aged < 10 years	COVID-19 affects healthy pediatricians more than pediatric patients
**Shen K et al [17]**	December 2019-January 2020	China	28 children aged from 1 month to 17 years	Diagnosis, treatment, and prevention of 2019 novel coronavirus infection in children
**Gkoutzourelas A et al [18]**	December 2019- June 2020	In the World	Infected children	Kawasaki Disease and COVID-19
**The Public Health England [19]**	May,7 -April 22, 2021	UK	ND	SARS-CoV-2 variants of concern and variants under investigation in England
**Torjesen I [20]**	May-June 2021	London, UK	ND	Covid-19: Delta variant is now UK's most dominant strain and spreading through schools
**Sheikh A et al [21]**	May-June 2021	Scotland	ND	SARS-CoV-2 Delta VOC in Scotland: demographics, risk of hospital admission, and vaccine effectiveness
**O'Dowd A [22]**	June 11-17, 2021	London, UK	58 830 infected patients	Covid-19: Cases of Delta variant rise by 79%, but rate of growth slows
**Torjesen I [23]**	May 2021 to 13 December 2021	London, UK	Infected children	Covid-19: Omicron variant is linked to steep rise in hospital admissions of very young children
**Scientific Advisory Group for Emergencies [24]**	6 January 2022	London, UK	Children aged < 18 years	Child admissions and severity
**Tian D et al [25]**	November – December 2021	In the World	ND	The emergence and epidemic characteristics of the highly mutated SARS-CoV-2 Omicron variant
**Callaway E and Ledford H [26]**	November - December 2021	South Africa	8,561 cases	Omicron variant: How bad is Omicron?
**McNiff S [27]**	December 26, 2021- January 1, 2022	US	Children aged between 5 and 17 years	Covid hospitalizations rising in kids too young for vaccine
**Viana R et al [28]**	December 20, 2021	South Africa	ND	Rapid epidemic expansion of the SARS-CoV-2 Omicron variant in southern Africa
**TSabouri S et al [29]**	February 26, 2020- June 10, 2020	In the World	Children under 18 years old	Risk Factors for Severity in Children with Coronavirus Disease 2019
**Mannheim J et al [30]**	March 5-April 8, 2020	Chicago, USA	64 children aged under 17 years	Characteristics of Hospitalized Pediatric Coronavirus Disease 2019 Cases
**Wu Z et al [31]**	February 2020	China	72 314 Cases	Characteristics of and Important Lessons from the Coronavirus Disease

				2019 (COVID-19) outbreak in China: summary of a report of 72314 cases from the Chinese center
**CDC [32]**	February 12-April 2, 2020	United States	2,572 children	Coronavirus Disease 2019 in Children
**Dong Y et al [33]**	January 16 - February 8, 2020	China	728 infected children	Epidemiology of COVID-19 Among Children in China
**CDC [34]**	March-December 2020	United States	Children aged under 17 years	Infections among children: Covid-19
**Melissa Borrelli et al [35]**	January-November 2020	Different countries	Pediatric population	Coronavirus Disease 2019 in Children
**Dong Y et al [36]**	January 16 - February 8, 2020	China	2135 pediatric patient	Epidemiological characteristics of 2143 pediatric patients with 2019 coronavirus disease
**Bhuiyan MU et al [37]**	Until June 4, 2020	In different countries	1,214 Children under 5 years	Epidemiology of COVID-19 infection in young children under five years
**Pan A et al [38]**	December 8, 2019, March 8, 2020	Wuhan, China	32 583 cases	Association of Public Health Interventions with the Epidemiology of the COVID-19 Outbreak
**Public Health Agency of Canada COVID-19 [39]**	January 15-July 9, 2020	Canada	106,804 cases	Descriptive epidemiology of deceased cases of COVID-19 reported during the initial wave of the epidemic
**Elimian KO et al [40]**	February27 – June 6, 2020	Nigeria	40 926 cases	Descriptive epidemiology of coronavirus disease 2019
**Pollán M et al [41]**	April 27 - May 11, 202	Spain	61 075 participants	Prevalence of SARS-CoV-2 in Spain (ENE-COVID): a nationwide, population-based seroepidemiological study
**Leidman E et al [42]**	March 1–December 12, 2020	United States	Children aged under 17 years	COVID-19 Trends Among Persons Aged 0-24 Years
**Alahmari AA et al [43]**	June 20, 2020	Saudi Arabia	240,474 patients	Epidemiological and clinical features of COVID-19 patients
**Nachega et al [44]**	March 10- July 31, 2020 2	Congo, south Africa	766 confirmed COVID-19 cases	Clinical Characteristics and Outcomes of Patients Hospitalized for COVID-19 in Africa
**Poustchi H et al [45]**	April 17- June 2, 2020	Iran	9181 individuals	SARS-CoV-2 antibody seroprevalence in the general population and high-risk occupational groups across 18 cities in Iran
**Jakhmola S et al [46]**	March-April, 2020	Different countries	ND	A comparative analysis of COVID-19 outbreaks on age groups and both the

				sexes of population from India and other countries
**Korea Disease Control and Prevention Agency [47]**	April 4, 2020	Korea	10,265 confirmed cases of COVID-19	Weekly report on the COVID-19 situation in the Republic of Korea
**Jeng MJ et al [48]**	April 6, 2020	Taiwan	12 Children	Coronavirus disease 2019 in children
**Sola AM et al [49]**	April- May 2020	United states	28 children	Prevalence of SARS-CoV-2 Infection in Children Without Symptoms of Coronavirus Disease 2019
**World Health Organization [50]**	January 17-23 2022	Six WHO regions	21 million new cases	COVID-19 weekly epidemiological update
**National Association of Head Teachers [51]**	27 May 2021	UK	ND	Data on covid-19 variant cases linked to schools
**Office for National Statistics [52]**	May 29, 2021	UK	Children aged above 2 years	Coronavirus (COVID-19) Infection Survey
**Dougherty K et al [53]**	April-May 2021	Oklahoma, United states	Children aged above 2 years	SARS-CoV-2 B.1.617.2 (Delta) Variant COVID-19 Outbreak Associated with a Gymnastics Facility
**Shoji K et al [54]**	October 2020-May 2021	Japan	Patients < 18 years	Comparison of the clinical characteristics and outcomes of COVID-19 in children before and after the emergence of Delta variant of concern in Japan
**Edward PR et al [55]**	October 15th, 2020-August 31st, 2021	USA	Patients < 18 years	Severity of Illness Caused by Severe Acute Respiratory Syndrome Coronavirus 2 Variants of Concern in Children
**Ryu BH et al [56]**	December 2020- August 2021	South Korea	235 patients	Features of COVID-19 Among Children and Adolescents Without Risk Factors Before and After the Delta Variant Outbreak
**Siegel DA et al [57]**	August 2020–August 2021	United states	Children aged <17 years	Trends in COVID-19 cases, emergency department visits, and hospital admissions among children and adolescents aged 0-17 years
**Wolter N et al [58]**	October 1 - December 6 2021	South Africa	161 328 cases of COVID-19	Early assessment of the clinical severity of the SARS-CoV-2 Omicron variant in South Africa.
**Sheikh A et al [59]**	November 1–December 19, 2021	Scotland, UK	23 840 cases	Severity of Omicron variant of concern and vaccine effectiveness

				against symptomatic disease: national cohort with nested test negative design study in Scotland
**WHO - MRC Centre for Global Infectious Disease Analysis [60]**	1st and 14th December 2021	England, UK	ND	Hospitalisation risk for Omicron cases in England
**Wang L et al [61]**	September 1-December 21, 2021	USA	577,938 cases	Comparison of outcomes from COVID infection in pediatric and adult patients before and after the emergence of Omicron.
**Lee B and Raszka WV Jr [62]**	March 10 - April 10, 2020	Geneva	Children <16 years	COVID-19 Transmission and Children: The Child Is Not to Blame
**Maltezou HC et al [63]**	February 26-May 32020	Greece	43 children	Transmission dynamics of SARS-CoV-2 within families with children in Greece
**Laws RL et al [64]**	March – May 2020	United states	68 children < 18 years	Symptoms and Transmission of SARS-CoV-2 Among Children
**Kitano T et al [65]**	December 2020	In the World	3,788 global pediatric COVID-19 deaths	The differential impact of pediatric COVID-19 between high-income countries and low- and middle-income countries
**Pascarella S et al [66]**	May 2021	Italy	ND	SARS-CoV-2 B.1.617 Indian variants: Are electrostatic potential changes responsible for a higher transmission rate?
**Loenenbach A et al [67]**	January–February 2021	Hesse, Germany	3 Child care centres	SARS-CoV-2 variant B.1.1.7 susceptibility and infectiousness of children and adults deduced from investigations of childcare centre outbreaks
**Singh J et al [68]**	January-April, 2021	India	ND	SARS-CoV-2 variants of concern are emerging in India.
**Callaway E [69]**	May-June 2021	UK, US, South Africa		Delta coronavirus variant: scientists brace for impact
**Schmidt F et al [70]**	July 2021	USA	ND	High genetic barrier to SARS-CoV-2 polyclonal neutralizing antibody escape
**CDC [71]**	2021 Dec 23	United states	ND	COVID Data Tracker
**Shen Q et al [72]**	January 8 - February 19, 2020	China	6 Children	Novel coronavirus infection in children outside of Wuhan, China
**Han Y et al [73]**	January 31-February 16, 2020	China	32 cases	A comparative-descriptive analysis of clinical characteristics in 2019-coronavirus-infected children and adults

**Ogata T et al [74]**	August-September, 2020	Japan	214 patients	Shorter Incubation Period among Unvaccinated Delta Variant Coronavirus Disease 2019 Patients in Japan
**Wang Y et al [75]**	May 21 and June 18, 2021	China	159 cases	Transmission, viral kinetics and clinical characteristics of the emergent SARS-CoV-2 Delta VOC in Guangzhou, China
**Zhang M et al [76]**	May–June 2021	China	167 cases	Transmission Dynamics of an Outbreak of the COVID-19 Delta Variant B.1.617.2 - Guangdong Province, China
**Li L et al [77]**	May - June 2021.	China	ND	Transmission and containment of the SARS-CoV-2 Delta variant of concern in Guangzhou, China:
**Jansen L et al [78]**	November-December, 2021	Nebraska, Unites states	1 patient	Investigation of a SARS-CoV-2 B.1.1.529 (Omicron) variant cluster
**Su L et al [79]**	January 24, 2020 - February 24, 2020	China	9 children	The different clinical characteristics of corona virus disease cases between children and their families in China—the character of children with COVID-19
**Feng K et al [80]**	January 16 February 6, 2020	China	15 children	Analysis of CT features of 15 children with 2019 novel coronavirus infection
**Ma X et al [81]**	January-March 2020	China	6 children	Do children need a longer time to shed SARS-CoV-2 in stool than adults?
**Hu Z et al [82]**	January 28 - February 9, 2020	China	24 cases	Clinical characteristics of 24 asymptomatic infections with COVID-19 screened among close contacts in Nanjing, China
**Li W et al [83]**	January 28-February 8, 2020	China	5 children	Chest computed tomography in children with COVID-19 respiratory infection
**Yang P et al [84]**	January – February 2020	China	416 children Under 10 years old	Corona Virus Disease 2019, a growing threat to children?
**Yu N et al [85]**	January 1 - February 8, 2020	China	7 patients	Clinical features and obstetric and neonatal outcomes of pregnant patients with COVID-19 in Wuhan, China
**Qiu H et al [86]**	January 17 - March 1, 2020	Zhejiang, China	36 children	Clinical and epidemiological features of 36 children with coronavirus disease 2019

				(COVID-19) in Zhejiang, China
**Zheng F et al [87]**	February 2020	China	25 pediatric cases	Clinical Characteristics of Children with Coronavirus Disease 2019 in Hubei, China
**Jeng M-J [88]**	March – April 2020	Taiwan	Children younger than 20 years of age	Coronavirus disease 2019 in children
**Xia W et al [89]**	January 23- February 8, 2020	China	20 pediatric cases	Clinical and CT features in pediatric patients with COVID-19 infection: different points from adults
**Wang D et al [90]**	January 25- February 21, 2020	China	31 children	Clinical analysis of 31 cases of 2019 novel coronavirus infection in children from six provinces (autonomous region) of northern China
**Wei M et al [91]**	December 8, 2019 - February 6, 2020	China	9 infants	Novel coronavirus infection in hospitalized infants under 1 year of age in China
**Liu W et al [92]**	January 7 - January 15, 2020	China	366 hospitalized children	Detection of COVID-19 in children in early January 2020 Wuhan, China
**Lu X et al [93]**	January 28 - February 26, 2020	China	1391 children	SARS-CoV-2 Infection in Children
**Parri N and Lenge M [94]**	March 3 - March 27, 2020	Italy	100 children younger than 18 years of age	Children with Covid-19 in Pediatric Emergency Departments in Italy
**Sun D et al [95]**	January 24 - February 24, 2020	China	8 children	Clinical features of severe pediatric patients with coronavirus disease 2019 in Wuhan
**Zhou Y et al [96]**	January 20- February 10, 2020	China	9 children, aged Under 3 years	Clinical features and chest CT findings of coronavirus disease 2019 in infants and young children
**Al-Beltagi M et al [97]**	April, 2021	In different countries	Children aged under 18 years	Pediatric gastrointestinal disorders in SARS-CoV-2 infection: Epidemiological and clinical implications
**Han YN et al [98]**	1st January - 16th February, 2020	China	7 children	A comparative-descriptive analysis of clinical characteristics in 2019-coronavirusinfected children and adults
**Aguila EJT et al [99]**	December – February, 2020	China, Hong Kong, and Singapore	2645 patients	COVID-19 and its effects on the digestive system and endoscopy practice
**Jin X et al [100]**	January 17- February 8, 2020	Zhejiang, China	74 patients	Epidemiological, clinical and virological characteristics of 74 cases of coronavirus-infected disease 2019 (COVID-19)

				with gastrointestinal symptoms.
**Jahangir M et al [101]**	December 1, 2019 - April 9, 2020	China, US	pediatric population aged under 19 years	Clinical manifestations and outcomes of COVID-19 in the pediatric population
**Pan L et al [102]**	January 18, 2020 - March 18, 2020	Hubei, China	204 Patients	Clinical characteristics of COVID-19 patients with digestive symptoms in Hubei, China
**Cheung KS et al [103]**	February 2 - February 29, 2020	Hong Kong, China	59 patients	Gastrointestinal manifestations of SARS-CoV-2 infection and virus load in fecal samples from the Hong Kong
**Yang X et al [104]**	December, 2019-February 9 2020	Wuhan, China	52 patients	Clinical course and outcomes of critically ill patients with SARS-CoV-2 pneumonia in Wuhan, China
**Chen N et al [105]**	January 1-25, 2020	Wuhan, China	99 patients	Epidemiological and clinical characteristics of 99 cases of 2019 novel coronavirus pneumonia in Wuhan, China
**Wang Y et al [106]**	January 11, 2020 - February 29, 2020	Shenzhen, China	55 Patients	Clinical outcome of 55 asymptomatic cases at the time of hospital admission infected with SARS-Coronavirus-2 in Shenzhen, China
**Xu X et al [107]**	January 10, 2020 – January, 26 2020	Zhejiang China	62 patients	Clinical findings in a group of patients infected with the 2019 novel coronavirus (SARS-Cov-2) outside of Wuhan, China
**Guan W et al [108]**	January 2020	China	1099 patients	Clinical characteristics of coronavirus disease 2019 in China
**Young B et al [109]**	January 23 - February 3, 2020	Singapore	18 patients	Epidemiologic features and clinical course of patients infected with SARS-CoV-2 in Singapore
**Han C et al [110]**	February 13 - February 29, 2020	China	206 patients	Digestive symptoms in COVID-19 patients with mild disease severity: Clinical presentation, stool viral RNA testing, and outcomes
**Giacomet V et al [111]**	February 21 - May 1, 2020	Italy	127 children	Gastrointestinal Symptoms in Severe COVID-19 Children
**Hu Z et al [112]**	April 2020-August 2021	Wuhan, China	677 patients	Differences in Clinical Characteristics Between Delta Variant and Wild-Type SARS-CoV-2 Infected Patients

**Italian National Health Institute [113]**	March 26, 2020	Italy	ND	Coronavirus epidemic: situation report
**Tagarro A et al [114]**	March 2 -16, 2020	Madrid, Spain	365 children	Screening and severity of coronavirus disease 2019 (COVID-19) in Children in Madrid, Spain
**Mahase E [115]**	May-June 2021	UK	ND	. Delta variant: What is happening with transmission, hospital admissions, and restrictions?
**B.-H. Ryu et al [116]**	December 2020 to August 2021	South Korea	235 patients	Features of COVID-19 Among Children and Adolescents Without Risk Factors Before and After the Delta Variant Outbreak in South Korea
**Wang L et al [117]**	December 26, 2021- January 6, 2022	United states	7,201 infected children	COVID infection severity in children under 5 years old before and after Omicron emergence in the US
**Sheikh A et al [118]**	April 1 to June 6, 2021	UK	19 543 confirmed SARS-CoV-2	SARS-CoV-2 Delta VOC in Scotland: demographics, risk of hospital admission, and vaccine effectiveness.
**Twohig KA et al [119]**	March 29 - May 23, 2021	UK	49 930 cases	Hospital admission and emergency care attendance risk for SARS-CoV-2 Delta (B.1.617.2) compared with alpha (B.1.1.7) variants of concern
**Murillo-Zamora E et al [120]**	March 2020-July 2021	Mexico, America	Children aged < 10 years	Decreased risk of COVID-19 pneumonia in children and adolescents during the Delta variant emergence
**Delahoy MJ et al [121]**	March 1, 2020–August 14, 2021	United states	Children aged under 17 years	Hospitalizations associated with COVID-19 among children and adolescents
**Cheng QR et al [122]**	January 25 - February 21, 2020<br>July 22 - August 20, 2021	China	23 children<br>66 children	Chest CT features of children infected by B.1.617.2 (Delta) variant of COVID-19.
**Brough HA et al [123]**	January 16 - February 8, 2020	China	2143 pediatric patients	Managing childhood allergies and immunodeficiencies during respiratory virus epidemics - the 2020 COVID-19 pandemic.
**Al Yazidi LS et al [124]**	2020 - 2021	Oman	140 patients	COVID-19 AND PRIMARY IMMUNODEFICIENCY

**Ahanchian H et al [125]**	March- April 2020	Iran	1 patient	COVID-19 in a Child with Primary Specific Antibody Deficiency
**CDC [126]**	April 2, 2020	United states	ND	Coronavirus Disease 2019 (COVID-19).
**Liu BM et al [127]**	December 2020	USA	ND	Role of Host Immune and Inflammatory Responses in COVID-19 Cases with Underlying Primary Immunodeficiency
**Babaha F et al [128]**	July 2020	Italy	9 Cases	Primary Immunodeficiency Diseases in COVID-19 Pandemic: A Predisposing or Protective Factor?
**Delavari S et al [129]**	December 2020	Iran	Children aged than 14 years	Impact of SARS-CoV-2 Pandemic on Patients with Primary Immunodeficiency
**Meyts I et al [130]**	March 16 - June 30, 2020	An international study	94 patients	Coronavirus disease 2019 in patients with inborn errors of immunity
**Shields AM et al [131]**	March – July, 2020	UK	100 patients	COVID-19 in patients with primary and secondary immunodeficiency
**Quinti I et al [132]**	2020 Apr 22	Italy	7 patients	A possible role for B cells in COVID-19? Lesson from patients with agammaglobulinemia
**Soresina A et al [133]**	During 2020	Northern Italy	2 patients	Two X-linked agammaglobulinemia patients develop pneumonia as COVID-19 manifestation but recover
**Kew OM et al [134]**	Sep 13, 2005	United states	ND	Vaccine-derived polioviruses and the endgame strategy for global polio
**Driss N et al [135]**	March 2006- August 2010	Tunis	82 patients	High susceptibility for enterovirus infection and virus excretion features in Tunisian patients with primary immunodeficiencies
**Driss N et al [136]**	October 2009 - April 2013	Tunis	1 patient	Sequential asymptomatic enterovirus infections in a patient with major histocompatibility complex class II primary immunodeficiency

**Lamari A et al [137]**	November 2013, July 2014	Tunis	35 samples from 1 patient	Iterative Excretion of Human Cosaviruses from Different Genotypes
**Avanzato VA et al [138]**	February- July 2020	USA	1 patient	Case Study: Prolonged Infectious SARS-CoV-2 Shedding from an Asymptomatic Immunocompromised Individual with Cancer
**Tarhini H et al [139]**	April 2020 – June 2020	Africa, Tunisia, European countries	3 patients	Long term SARS-CoV-2 infectiousness among three immunocompromised patients: from prolonged viral shedding to SARS-CoV-2 superinfection
**Hadjadj J et al [140]**	January - April 2021	France	77 cases	Immunogenicity of BNT162b2 vaccine against the Alpha and Delta variants in immunocompromised patients with systemic inflammatory diseases
**Chappell H et al [141]**	March 2020 - 2021	UK	1527 participants	Immunocompromised children and young people are at no increased risk of severe COVID-19
**WHO [142]**	26 November 2021	South Africa	ND	Classification of Omicron (B.1.1.529): SARS-CoV-2 Variant of Concern
**CDC [143]**	Mar. 29, 2022	United states	ND	Omicron Variant
**Wang L and Cheng G [144]**	November - December 2021	In the World	ND	Sequence analysis of the emerging SARS-CoV-2 variant Omicron in South Africa
**Bansal N et al [145]**	2021-2022	India	ND	SARS-CoV-2 variants in immunocompromised COVID-19 patients: The underlying causes and the way forward
**Gao S-J et al [146]**	Since November 2021	In the world	ND	Omicron variant (B.1.1.529) of SARS-CoV-2, a global urgent public health alert!
**Singhal T [147]**	November, December, 2021	India	ND	The Emergence of Omicron: Challenging Times Are Here Again
**Fang et al [148]**	Until October 2020	In different countries	ND	Kawasaki like Illness Due to COVID-19
**Ozen S et al [149]**	June 2005	Different countries	ND	EULAR/PReS endorsed consensus criteria for the classification of childhood vasculitides
**B.W. McCrindle et al [150]**	2011- 2017	In different countries	ND	Diagnosis, Treatment, and long-term management of Kawasaki disease: a scientific statement for health professionals from

				the American heart association
**Kawasaki T et al [151]**	In 1974	Japan	ND	A new infantile acute febrile mucocutaneous lymph node syndrome (MLNS) prevailing in Japan
**Kato H et al [152]**	1973 - 1983	Japan	594 children	Long-term consequences of Kawasaki disease. A 10- to 21-year follow-up study of 594 patients
**Xu S et al [153]**	May 2020	China, UK, USA, Italy	ND	COVID-19 and Kawasaki disease in children
**Kanegaye JT et al [154]**	January 2001 - August 2006	United states	373 patients	Recognition of a Kawasaki disease shock syndrome
**Dietz SM et al [155]**	2017	In different countries	Children under 5 years	Dissecting Kawasaki disease
**McCrindle BW et al [156]**	2017	North America, Asia	ND	Diagnosis, treatment, and long-term management of Kawasaki disease
**L. Verdoni et al [157]**	January 1, 2015 - April 20, 2020	Italy	29 patients	An outbreak of severe Kawasaki-like disease at the Italian epicenter of the SARS-CoV-2 epidemic
**Saguil A et al [158]**	November 2013 - September 2014	United States	ND	Diagnosis and management of Kawasaki disease
**Kabeerdoss J et al [159]**	December 1, 2019—August 31, 2020	In different countries	Children aged below 3 years	Severe COVID-19, multisystem inflammatory syndrome in children, and Kawasaki disease: immunological mechanisms, clinical manifestations and management
**Toubiana J et al [160]**	April 27 - May 15, 2020	France	21 children and adolescent	Kawasaki-like multisystem inflammatory syndrome in children during the covid-19 pandemic in Paris, France: prospective observational study
**Jones VG et al [161]**	January 2020	California, United states	1 child	COVID-19 and Kawasaki disease: novel virus and novel case
**Rivera-Figueroa E et al [162]**	May 2020	USA	1 patient	Incomplete Kawasaki disease in a child with COVID-19
**Pouletty M et al [163]**	Since April 2020	Paris, France	16 patients	Pediatric multisystem inflammatory syndrome temporally associated with SARS-CoV-2 mimicking Kawasaki disease (Kawa-COVID-19)
**Ramcharan T et al [164]**	April 10 - May 9, 2020	African/Afro-Caribbean, South Asian	15 children	Pediatric inflammatory multisystem syndrome: temporally associated

				with SARSCoV-2 (PIMS-TS)
**Ouldali N et al [165]**	July 2020	Barcelona, Spain	12 children	Response to: 'Correspondence on 'Pediatric multisystem inflammatory syndrome temporally associated with SARS-CoV-2 mimicking Kawasaki disease (Kawa-COVID-19)
**Lee PY et al [166]**	March - June 2020	USA	28 patients	Distinct clinical and immunological features of SARS-COV-2-induced multisystem inflammatory syndrome in children
**Pino R et al [167]**	March 23 - May 14, 2020	Barcelona, Spain	Patients aged >12 years	Correspondence on: 'Pediatric multisystem inflammatory syndrome temporally associated with SARS-CoV-2 mimicking Kawasaki disease (Kawa-COVID-19)
**Jones VG et al [168]**	June 2020	California United states	1 patient	COVID-19 and Kawasaki disease: novel virus and novel case.
**Thompson LA et al [169]**	June 2021	Florida, United states	Children aged 12 years and older	Return to Play After COVID-19 Infection in Children
**Centers for Disease Control and Prevention [170]**	April 26, 2020	UK	8 Cases	Emergency preparedness and response: multisystem inflammatory syndrome in children (MIS-C) associated with coronavirus disease 2019 (COVID-19)
**Nakra N et al [171]**	February 18 - April 20, 2020	UK, Italy, France, USA and Switzerland	70 children	Multi-System Inflammatory Syndrome in Children (MIS-C) Following SARS-CoV-2 Infection
**Riollano-Cruz M et al [172]**	24 April 19 June 2020	New York, US	15 Patients	Multisystem Inflammatory Syndrome in Children (MIS-C) Related to COVID-19
**Feldstein LR et al [173]**	March 15 - May 20, 2020	United states	Patients aged < 21 years	Multisystem Inflammatory Syndrome in U.S. Children and Adolescents.
**Miller J et al [174]**	April 18 - May 22, 2020	Columbia, South America	44 patients	Gastrointestinal symptoms as a major presentation component of a novel multisystem inflammatory syndrome in children (MIS-C) that is related to COVID-19
**Chiotos K et al [175]**	April-May, 2020	UK, USA	6 Children	Multisystem Inflammatory Syndrome in Children During the

				Coronavirus 2019 Pandemic
**Grimaud M et al [176]**	April 15 - April 27, 2020	Paris, France	20 children	Acute myocarditis and multisystem inflammatory emerging disease following SARS-CoV-2 infection in critically ill children.
**Belhadjer Z et al [177]**	March 22 - April 30, 2020	France and Switzerland	35 children	Acute heart failure in multisystem inflammatory syndrome in children (MIS-C) in the context of global SARS-CoV-2 pandemic
**Christensen PA et al [178]**	March 15 - September 20, 2021	Houston, US	16,965 patients	Delta Variants of SARS-CoV-2 Cause Significantly Increased Vaccine Breakthrough COVID-19 Cases in Houston, Texas
**Oualha M et al [179]**	February 10 - April 20, 2020	Paris, France	27 Children	Severe and fatal forms of COVID-19 in children
**De Jacobis IT et al [180]**	February - July 2020	Rome, Italy	66 patients	Clinical characteristics of children infected with SARS-CoV-2 in Italy
**Pecoraro L et al [181]**	April 16, 2020	Italy	1123 children	The psychophysical impact that COVID-19 has on children must not be underestimated
**Hoffmann M et al [182]**	Mar 5, 2020	Germany	ND	SARS-CoV-2 cell entry depends on ACE2 and TMPRSS2 and is blocked by a clinically proven protease inhibitor
**Wang Q et al [183]**	February 24, 2020	China	77,262 cases	Structural and Functional Basis of SARS-CoV-2 Entry by Using Human ACE2
**Lee PI et al [184]**	February 11, 2020	Taiwan	44,672 confirmed cases	Are children less susceptible to COVID-19?
**Be´ne´teau-Burnat B et al [185]**	1990 February	France	187 Children	Serum angiotensin-converting enzyme in healthy and sarcoidotic children: comparison with the reference interval for adults
**Kunling Shen et al [186]**	January 30, 2020	China	9692 confrmed cases	Diagnosis, treatment, and prevention of 2019 novel coronavirus infection in children: experts' consensus statement.
**Gold JE et al [187]**	January 2020 - September 2020	USA	50 subjects	Analysis of Measles-Mumps-Rubella (MMR) Titers of Recovered COVID-19 Patients
**Escobar LE et al [188]**	April 20, 2020	United states	ND	BCG vaccine protection from severe coronavirus disease 2019 (COVID-19)

**Haddad-Boubaker S et al [189]**	December 8, 2019 - January 15, 2021	Tunis	14 similar segments	In silico comparative study of SARS-CoV-2 proteins and antigenic proteins in BCG, OPV, MMR and other vaccines: evidence of a possible putative protective effect
**Bandi S et al [190]**	March 12 - April 20, 2020	Chicago, United states	474 children	African American children are at higher risk for COVID-19 infection
**DeBiasi RL et al [191]**	March 15 April 30, 2020	Washington, United states	177 children	Severe COVID-19 in children and young adults in the Washington
**Whittaker E et al [192]**	March 23 - May 16, 2020	UK	58 children	Clinical characteristics of 58 children with a pediatric inflammatory multisystem syndrome temporally associated with SARS-CoV-2
**Onder G et al [193]**	February 20, 2020	Italy	ND	Case-fatality rate and characteristics of patients dying in relation to COVID-19 in Italy
**Shekerdemian LS et al [194]**	March 14 - April 3, 2020	North America, United states	48 Children	Characteristics and Outcomes of Children with Coronavirus Disease 2019 (COVID-19) Infection Admitted to US and Canadian Pediatric Intensive Care Units
**Zhou F et al [195]**	December 29, 2019 - January 31, 2020	China	191 patients	Clinical course and risk factors for mortality of adult inpatients with COVID-19 in Wuhan, China: a retrospective cohort study
**Shi B et al [196]**	February 3, 2020	China	1 case	Severe Pneumonia Due to SARS-CoV-2 and respiratory syncytial virus infection: a case report
**Aleem A et al [197]**	December 2021 - January 2022	In the world	ND	Emerging Variants of SARS-CoV-2 And Novel Therapeutics Against Coronavirus (COVID-19).
**Galloway SE et al [198]**	December 29, 2020–January 12, 2021	United states	ND	Emergence of SARSCoV-2 B.1.1.7 lineage
**Shiehzadegan S et al [199]**	Since July 2021	USA	ND	Analysis of the Delta Variant B.1.617.2 COVID-19
**Sanches PRS et al [200]**	Sep 2021	UK, South Africa, Brazil and India	ND	Recent advances in SARS-CoV-2 Spike protein and RBD mutations comparison between new variants Alpha (B.1.1.7, United Kingdom)
**He X et al [201]**	October 2021	China	ND	The challenges of COVID-19 Delta variant:

				Prevention and vaccine development
**Hesman Saey. T, Garcia de Jesús. E [202]**	October-December, 2021	United states	ND	Why the coronavirus's delta variant dominated 2021
**He X et al [203]**	November 26, 2021	China	ND	SARS-CoV-2 Omicron variant: Characteristics and prevention
**GISAID [204]**	December 7, 2021	Germany	ND	Variant : 21K (Omicron)
**Sun Y et al [205]**	October 2020-November 2021	China	ND	Origin and evolutionary analysis of the SARS-CoV-2 Omicron variant
**CDC [206]**	June 24, 2022	United states	Children aged under 17 years	COVID-19 Vaccine Recommendations for Children and Teens
**Ladhani SN [207]**	December, 2021-May 2022-ferbuary 2022	UK	Children aged between 5 and 11 years old	COVID-19 vaccination for children aged 5–11 years
**CDC [208]**	July 14, 2022	United states	ND	Pfizer-BioNTech COVID-19 Vaccine: Overview and Safety
**WHO [209]**	10 June 2022		ND	The Pfizer BioNTech (BNT162b2) COVID-19 vaccine: What you need to know
**CDC [210]**	July 19, 2022	United states	Children aged: under 11 years<br>12-17 years	Stay Up to Date with Your COVID-19 Vaccines-Moderna
**Quentin Nogues et al [211]**	28 juillet 2021	France	Children aged12-17 years old	Covid-19 : le vaccin Spikevax® de Moderna peut être utilisé à partir de l'âge de 12 ans
**Ian Smith [212]**	May 2021-February 2022		Children aged 5-17 years	COVID vaccines: Which countries are vaccinating children over 5 and how do they compare?
**Woodworth KR et al [213]**	November 5, 2021	United states	Children aged between 5 and 11 years	The Advisory Committee on Immunization Practices' Interim Recommendation for Use of Pfizer-BioNTech COVID-19 Vaccine in Children Aged 5–11 Years — United States
**Dorabawila V et al [214]**	December 13, 2021 - January 30, 2022	United state	852,384 vaccinated children 12-17 years	Effectiveness of the BNT162b2 vaccine among children 5-11 and 12-17 years in New York after the Emergence of the Omicron Variant
**Sacco C et al [215]**	Jan 17 - April 13, 2022	Italy	1063035 vaccinated children aged 5-11	Effectiveness of BNT162b2 vaccine against SARS-CoV-2 infection and severe

				COVID-19 in children aged 5–11 years in Italy
**Zimmermann P et al [216]**	2021	Australia	Children under 12 years	Should children be vaccinated against COVID-19?
**Allo Docteur+-Eurs Africa [217]**	December 14, 2021	Tunis	Children aged 5-11 years	La Tunisie autorise le vaccin anti-Covid de Pfizer pour les enfants de 5 à 11 ans
**Thompson LA and Rasmussen SA [218]**	June 4, 2021	Florida, United States	ND	Children and COVID-19 Vaccines
**Zou X et al [219]**	Since May, 2021	United states	Children aged under 12 years	COVID-19 vaccines for children younger than 12 years: are we ready?
**Léa MORILLON [220]**	April-June 2021	France		Covid-19. États-Unis, Allemagne, Israël…Ces pays où la vaccination des ados a déjà commencé
**Ministry of Health and prevention [221]**	May-November, 2021	United Arab Emirates		COVID-19 vaccine for children
**Mallapaty S et al [222]**	November, 2021	United states, Europe, UK, United, Arab Emirates		How COVID vaccines shaped 2021 in eight powerful charts
**MEED Middle East busniss intelligence [223]**	March 2021-April 2022	Middle East and North Africa		Latest on the region's Covid-19 recovery
**Cassandra Willyard [224]**	June 2022	United states	Children aged under 5 years	FDA authorizes COVID vaccines for the littlest kids: what the data say
**Khatatbeh M [225]**	15-November-13-December 2021	eight countries, namely, Iraq, Jordan, Kuwait, Lebanon, Palestine, Qatar, Saudi Arabia (KSA), and the United Arab Emirates (UAE)	Children aged under 18 years	Children's rates of COVID-19 vaccination as reported by parents, vaccine hesitancy, and determinants of COVID-19 vaccine uptake among children: a multi-country study from the Eastern Mediterranean Region
**Sam-Agudu NA et al [226]**	December 2021	Africa countries	Children aged under 18 years	Children and adolescents in African countries should also be vaccinated for COVID-19
**Gerber JS [227]**	November 2021	United states	Children aged above 5 years	COVID-19 vaccines for children
**Hause AM et al [228]**	November 3–December 19, 2021	United states	Children aged above 5 years	Safety of COVID-19 Vaccination in United States Children Ages 5 to 11 Years.

**Kamidani S et al [229]**	February 2021	United states	Children of all ages	COVID-19 vaccine development: a pediatric perspective
**Walter EB et al [230]**	September 6, 2021	UK	Children aged under 12 years	Evaluation of the BNT162b2 Covid-19 Vaccine in Children 5 to 11 Years of Age

ND: Not determined
